# Knockdown of YY1 Inhibits *XIST* Expression and Enhances Cloned Pig Embryo Development

**DOI:** 10.3390/ijms232314572

**Published:** 2022-11-23

**Authors:** Yazheng Dong, Xiao Wu, Xitong Peng, Liusong Yang, Baohua Tan, Huaxing Zhao, Enqin Zheng, Linjun Hong, Gengyuan Cai, Zhenfang Wu, Zicong Li

**Affiliations:** 1 National Engineering Research Center for Breeding Swine Industry, South China Agricultural University, Guangzhou 510030, China; 2 Department of Animal Genetics, Breeding and Reproduction, College of Animal Science, Guangzhou 510030, China; 3 Guangdong Provincial Key Laboratory of Agro-Animal Genomics and Molecular Breeding, South China Agricultural University, Guangzhou 510030, China; 4 Guangdong Provincial Laboratory of Lingnan Modern Agricultural Science and Technology, Guangzhou 510642, China

**Keywords:** cloned pig embryos, X chromosome inactivation, YY1, *XIST*

## Abstract

The technique of cloning has wide applications in animal husbandry and human biomedicine. However, the very low developmental efficiency of cloned embryos limits the application of cloning. Ectopic *XIST*-expression-induced abnormal X chromosome inactivation (XCI) is a primary cause of the low developmental competence of cloned mouse and pig embryos. Knockout or knockdown of *XIST* improves cloning efficiency in both pigs and mice. The transcription factor Yin yang 1(YY1) plays a critical role in XCI by triggering the transcription of X-inactive specific transcript *(XIST)* and facilitating the localization of *XIST* RNA on the X chromosome. This study aimed to investigate whether RNA interference to suppress the expression of YY1 can inhibit erroneous *XIST* expression, rescue abnormal XCI, and improve the developmental ability of cloned pig embryos. The results showed that YY1 binds to the 5′ regulatory region of the porcine *XIST* gene in pig cells. The microinjection of YY1 siRNA into cloned pig embryos reduced the transcript abundance of *XIST* and upregulated the mRNA level of X-linked genes at the 4-cell and blastocyst stages. The siRNA-mediated knockdown of YY1 altered the transcriptome and enhanced the in vitro and in vivo developmental efficiency of cloned porcine embryos. These results suggested that YY1 participates in regulating *XIST* expression and XCI in cloned pig embryos and that the suppression of YY1 expression can increase the developmental rate of cloned pig embryos. The present study established a new method for improving the efficiency of pig cloning.

## 1. Introduction

The technique of cloning, also known as somatic cell nuclear transfer (SCNT), is used to propagate superior livestock [1,2], preserve endangered animals [3,4,5,6], generate cloned human embryos for isolation of therapeutic embryonic stem cells [7,8,9,10], and produce multi-purpose genetically engineered animals [11,12,13]. Thus, cloning is a powerful tool that has wide applications in animal production, life science, and biomedicine. Nevertheless, the efficiency of SCNT embryos in developing to term is very low, at about 2% [14,15] in mice and 1% in pigs [16]. Such a low developmental rate of full-term SCNT embryos limits the application and development of the SCNT technique.

Ectopic *XIST* expression induces abnormal silence of a part of X-linked genes, which resembles X chromosome inactivation (XCI); it is one of the primary causes of the low developmental competence of cloned mouse and pig embryos. Knockout of the *XIST* gene or knockdown of *Xist* expression not only rescued aberrant XCI but also resulted in an eight—ten-fold increase in the full-term developmental efficiency of cloned mouse embryos [17,18]. The injection of anti-*XIST* small interfering RNA (siRNA) or the short hairpin RNA (shRNA) plasmid and the inactivation of the *XIST* gene also increased the expression of X-linked genes and promoted the development of cloned pig embryos [19,20,21]. In addition, RNA interference (RNAi) of RLIM, a *XIST* transcription inducer, inhibited the expression of *XIST* and improved the developmental efficiency of cloned pig embryos [16].

Transcription factor YY1 plays key roles in regulating *XIST* functions. YY1 can activate *XIST* transcription and XCI in mouse and human cells by binding to the 5′ regulatory region of the *XIST* gene [22,23]. YY1 can also bind to *XIST* RNA to facilitate its localization on the X chromosome, thus promoting XCI in mouse and human cells [23,24,25].

We hypothesized that YY1 is involved in mediating the aberrant activation of *XIST* expression and XCI in cloned pig embryos. To test this hypothesis, we injected YY1 siRNA into cloned pig embryos to suppress YY1 expression and investigated its effects on *XIST* expression, XCI, and cloned pig embryo development. We demonstrated that siRNA-mediated knockdown of YY1 expression can suppress *XIST* expression, upregulate the expression of X-linked genes, and improve the developmental efficiency of cloned pig embryos.

## 2. Results

### 2.1. YY1 siRNA Transfection Inhibited the mRNA Expression of YY1 and XIST in Porcine Fibroblasts

**Figure 1:**
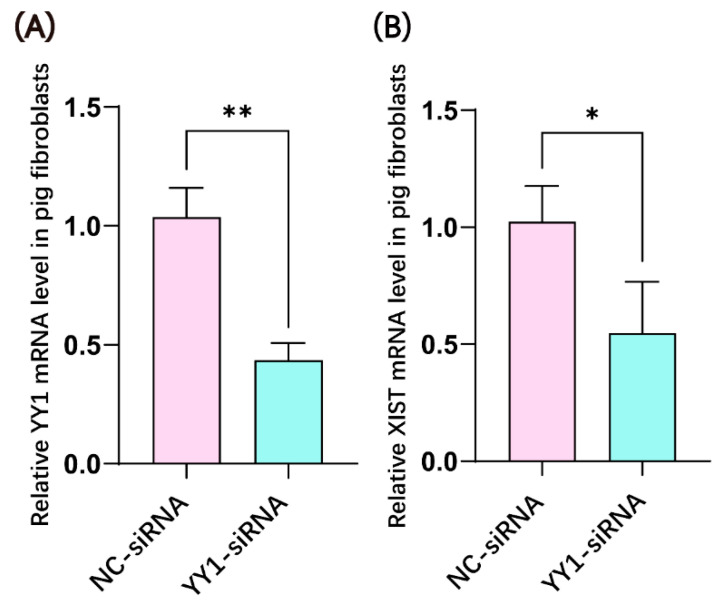
YY1 siRNA-mediated downregulation of *YY1* and *XIST* mRNA expression in female pig fibroblasts. (**A**) Knockdown of *YY1* expression by YY1 siRNA. (**B**) Inhibition of *XIST* expression via *YY1* expression knockdown. * and ** represent a significant difference at *p* < 0.05 and *p* < 0.01, respectively, where *n* = 3 in each group.

YY1 siRNA was transfected into porcine female fibroblasts to examine its effects on *YY1* and *XIST* mRNA expression. YY1 siRNA not only significantly decreased the level of *YY1* transcript (Figure 1A) but also significantly down-regulated *XIST* mRNA expression (Figure 1B).

### 2.2. YY1 Can Bind to the 5′ Regulatory Region of the Porcine XIST Gene

**Figure 2:**
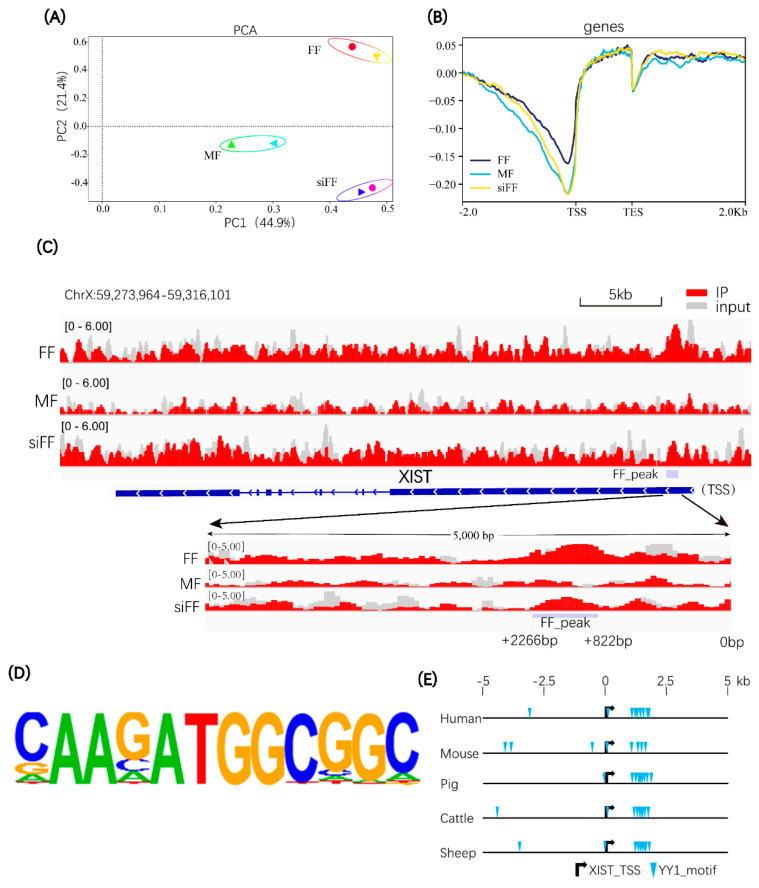
Analysis of the binding sites of YY1 on *XIST* Gene by Chip-seq. (**A**) Principal component analysis (PCA) plot. MF: male fibroblasts; FF: female fibroblasts; siFF: female fibroblasts transfected with YY1-siRNA. (**B**) Enrichment of YY1 binding sites within 2 kb upstream and 2 kb downstream of the transcription start site (TSS) and transcription termination site (TES) in the genome. The y-axis denotes the average log2 fold change relative to the control (**C**) Chip-seq revealed YY1 binding sites on the pig *XIST* gene. The white arrow in the blue box indicates the direction of transcription of the *XIST* gene. (**D**) Porcine YY1 DNA binding consensus motif. (**E**) Predicted YY1 binding sites on the *XIST* gene of different species.

To investigate whether YY1 activates porcine *XIST* transcription by binding to its regulatory region, we performed ChiP-seq analysis (for YY1) of three groups of pig cells, including male fibroblasts (MF), female fibroblasts (FF), and female fibroblasts transfected with YY1 siRNA (siFF). We hypothesized that in MF cells, the X-linked *XIST* gene should be silent to maintain the activation of the single X chromosome; in FF cells, one *XIST* allele should be expressed to maintain random XCI, and in siFF cells, *XIST* expression should be inhibited by transfected YY1 siRNA. The PCA results showed a distinct clustering of the samples from three groups (Figure 2A). The binding site analysis showed that YY1 had a central binding peak near the transcription start site (TSS) of genome-wide genes (Figure 2B). The analysis of the binding site of YY1 on the porcine *XIST* gene revealed the YY1 binding peak at the 5′ regulatory region (between +822 bp to +2266 bp) of the pig *XIST* gene in the FF cells, but this was not observed in the MF and siFF cells (Figure 2C). The DNA binding motif of YY1 was predicted by HOMER software analysis (Figure 2D). This motif was used to analyze the potential binding sites of YY1 on the *XIST* gene in human, mouse, pig, cattle, and sheep. In all the five analyzed mammalian species, the *XIST* gene contained similar clustered YY1 binding sites in the 5′ regulatory region (Figure 2E), implying that the binding of YY1 to the 5′ regulatory region of the *XIST* gene is conserved in mammals.

### 2.3. Microinjection of YY1 siRNA Inhibited the Expression of the YY1 Protein and Improved the Developmental Ability of Cloned Pig Embryos

**Figure 3:**
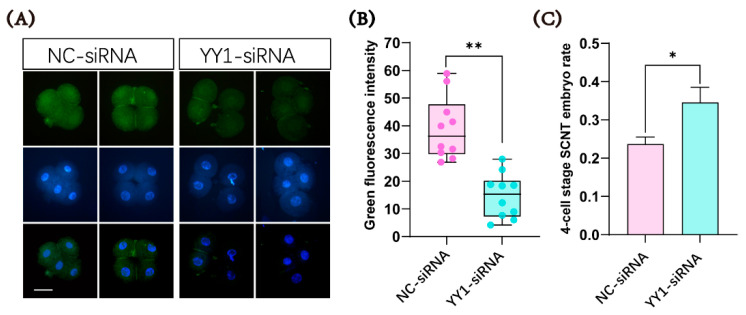
The effects of YY1-siRNA microinjection on YY1 protein expression and 4-cell-stage embryo rate of cloned pig embryos. (**A**) Immunofluorescence comparison of YY1 protein abundance between YY1-siRNA and NC-siRNA groups. Scale bar = 50 μm (**B**) YY1 protein quantification in YY1-siRNA and NC-siRNA groups (*n* = 10 in each group). (**C**) Comparison of 4-cell-stage embryo rate between YY1-siRNA and NC-siRNA groups (*n* = 4 in each group). * and ** represent a significant difference at *p* < 0.05 and *p* < 0.01, respectively.

YY1 siRNA was microinjected into cloned pig embryos to examine whether it could inhibit the expression of the YY1 protein. The immunofluorescence analysis showed that the level of the YY1 protein in the cloned pig embryos injected with YY1 siRNA was indeed significantly lower than that in the control group at the 4-cell stage (Figure 3A,B). When we collected cloned embryos at the 4-cell stage for immunofluorescence analysis, we also observed that the ratio of 4-cell-stage cloned embryos in the YY1 siRNA-injected group was significantly higher than that in the control group (Figure 3C)

To further study the effects of YY1 expression knockdown on the development of cloned pig embryos, we injected 10 µM or 50 µM of YY1 siRNA into cloned pig embryos. We found that the microinjection of 50 µM YY1 siRNA significantly increased the cleavage rate and blastocyst rate of the injected cloned embryos. In contrast, no significant effect was noticed on cloned pig embryo development after the injection of 10 µM YY1 siRNA (Table 1). This positive influence of 50 µM of YY1 siRNA on increasing the developmental competence of the cloned pig embryos was subsequently confirmed with more replicates and a larger number of injected embryos (Table 2). Moreover, 50 µM YY1 siRNA also improved the in vivo full-term developmental efficiency of the cloned pig embryos (Table 3).

### 2.4. Microinjection of YY1 siRNA Altered the Transcriptome of Cloned Pig Embryos

**Figure 4:**
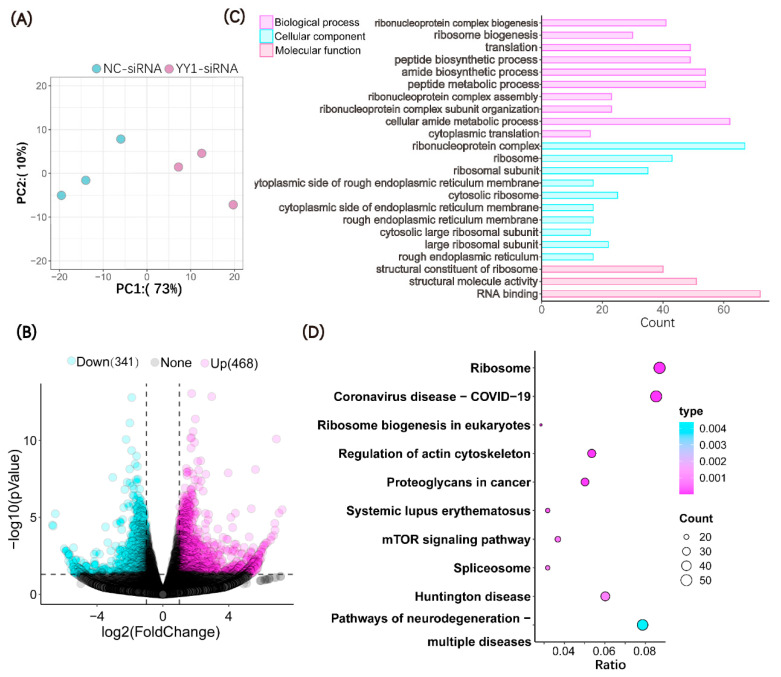
Transcriptome analysis of the 4-cell-stage control and YY1-knockdown SCNT embryos. (**A**) Principal component analysis (PCA) of two groups of cloned embryos. (**B**) Volcano plot of differentially expressed genes (DEGs) between two groups of cloned embryos. (**C**) The significantly enriched Gene Ontology (GO) terms of DEGs. The top 10 GO terms were shown in the biological process (BP) and cellular component (CC) categories. Only three GO terms were enriched in the category of molecular function (MF). (**D**) The Kyoto Encyclopedia of Genes and Genomes (KEGG) enrichment analysis of DEGs. The top 10 KEGG pathways are shown.

To investigate the effects of the injection of YY1 siRNA on the gene expression patterns of pig cloned embryos, we collected 4-cell-stage control embryos and YY1-knockdown embryos for transcriptome sequencing. According to the PCA results, the samples of the YY1-siRNA-injection group were separated from those of the NC-siRNA-injection group (Figure 4A), suggesting the different gene expression patterns of the two groups of embryos. A total of 809 differentially expressed genes (DEGs) were detected in the two groups of SCNT embryos; of these, 341 were down-regulated and 468 were upregulated in the YY1-siRNA-injected group relative to the control group (Figure 4B).

The DEGs identified by RNA sequencing were subjected to GO and KEGG analysis. The analysis of the biological process (BP), cellular component (CC), and molecular function (MF) based on the GO database showed that the DEGs were primarily enriched in ribonucleoprotein complex biogenesis, ribosome biogenesis, translation, ribonucleoprotein complex, ribosome, ribosomal subunits, structural constituent of ribosome, structural molecular activity, and RNA binding, etc. (Figure 4C). The KEGG analysis revealed that the DEGs between two groups of embryos were mainly enriched in the ribosome, Coronavirus disease, Huntington’s disease, etc. (Figure 4D).

### 2.5. Microinjection of YY1 siRNA Inhibited XIST Expression and Upregulated the mRNA Levels of X-Linked Genes in Cloned Pig Embryos

**Figure 5:**
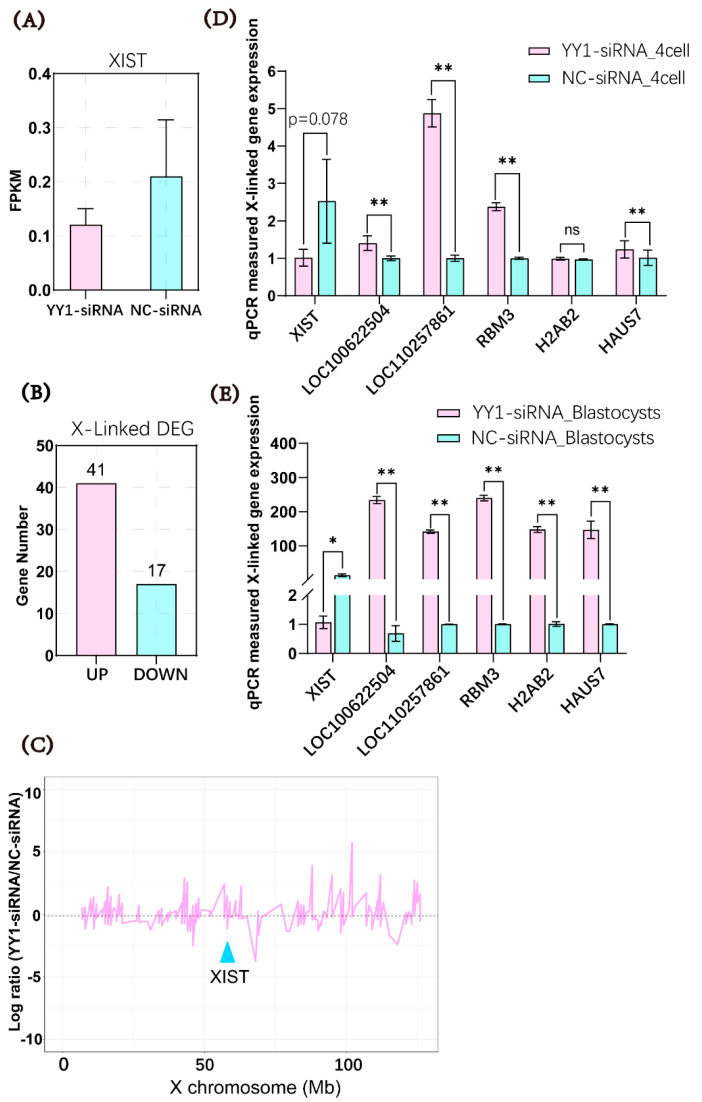
Effects of YY1 siRNA on the expression of *XIST* and X-linked genes in cloned embryos. (**A**) Level of *XIST* in YY1-siRNA group and NC-siRNA group measured by RNA-seq. (**B**) The number of X-linked differentially expressed genes in the YY1-siRNA group (vs. NC-siRNA group) detected by RNA-seq (*n* = 3). (**C**) Relative expression level of all the RNA-seq-detected X-linked genes of YY1-siRNA group (relative to the NC-siRNA group) plotted on the positions of the X chromosome. (**D**) Quantitative polymerase chain reaction (qPCR) used to measure the expression of *XIST* mRNA and X-linked genes in 4-cell-stage SCNT embryos. (**E**) qPCR used to measure mRNA expression of *XIST* and X-inked genes in blastocyst-stage SCNT embryos, * and ** represent a significant difference at *p* < 0.05 and *p* < 0.01, respectively. *n* = 3 in each group in (**A**–**E**).

The transcriptomic data analysis of the two groups of embryos showed that YY1 siRNA tended to inhibit the expression of *XIST* in the cloned embryos at the 4-cell stage (Figure 5A). The number of upregulated X-linked DEGs (41) in the YY1-siRNA-injection group was much higher than the number of downregulated X-linked DEGs (17) (Figure 5B). Furthermore, the analysis of the relative expression level of all the RNA-seq-detected X-linked genes of YY1-siRNA group indicated that expression of most of the detected X-linked genes were upregulated in the YY1-siRNA-injected embryos compared with control embryos (Figure 5C). These findings suggested that the RNAi of *YY1* favored the activation of the X chromosome in the cloned pig embryos. The influence of the knockdown of *YY1* on *XIST* and X-linked gene expression was further investigated by performing qPCR to examine the mRNA level of the *XIST* gene and five randomly selected X-linked genes in the two groups of cloned embryos at the 4-cell and blastocyst stages. The mRNA expression of *XIST* was almost significantly downregulated (*p* = 0.078), and the transcript levels of the four tested X-linked genes were significantly increased in the 4-cell stage of embryos with *YY1* knockdown compared with the control embryos (Figure 5D). At the blastocyst stage, the mRNA abundance of *XIST* decreased, and that of the five randomly selected X-linked genes was significantly elevated in the YY1-siRNA-injected embryos compared with the control embryos (Figure 5E).

## 3. Discussion

YY1 can activate *XIST* transcription and thereby induce XCI via binding to the 5′ regulatory region of the *XIST* gene [22,23]. YY1 can also facilitate *XIST* RNA localization to the X chromosome, thereby promoting XCI by binding to *XIST* RNA [23,24,25]. Therefore, YY1 possibly regulates XCI by either modulating *XIST* transcription or controlling the anchoring of *XIST* RNA on the X chromosome, or by both processes in different tissues at different developmental stages, or under different circumstances. In the cloned pig embryos injected with YY1 siRNA, the expression of X-linked genes was significantly upregulated at both the 4-cell stage and blastocyst stage, while the *XIST* mRNA level was only significantly decreased at the blastocyst stage but not at the 4-cell stage. This implies that in cloned pig embryos, YY1 may modulate X-linked gene expression at the 4-cell stage mainly by regulating the localization of *XIST* RNA, but the modulation at the blastocyst stage occurs mainly by regulating the transcription of the *XIST* gene.

The genes coding for many ribosomal proteins contain multiple YY1 binding sites in their promoter regions [26,27]. This suggests that YY1 can regulate the expression of ribosomal proteins and thereby modulate ribosome-related pathways. The GO and KEGG pathway analysis of the DEGs between the control and YY1-siRNA-injected embryos in this study showed that the DEGs were mainly enriched in ribosome-related pathways. This indicated that in addition to inhibiting *XIST* expression and promoting X-linked gene expression, the knockdown of YY1 expression in the cloned pig embryos also affected ribosome-related pathways. However, whether the changes in the ribosome-related pathways induced by RNAi of YY1 had a positive impact, a negative impact, or no impact on the development of the cloned pig embryos remains to be further examined.

In human and mouse cells, YY1 regulates XCI by binding to *XIST* DNA and RNA [22,23,24,25]. This study also demonstrated that YY1 might regulate XCI in pig cells and cloned embryos by binding to *XIST* DNA and RNA. The present study also indicated that the binding region of YY1 on the porcine *XIST* gene was similar to that on the human and mouse *XIST* gene, which is located at about +1500 bp downstream of the *XIST* transcription start site [22,23,25]. These data suggest that the regulatory mechanism of YY1 on *XIST* functions may be conserved in mammals.

We previously demonstrated that the siRNA-mediated knockdown of RLIM, another *XIST* transcription activator, also suppressed *XIST* expression and improved the developmental rate of cloned pig embryos [16]. However, RNA interference of RLIM exhibited only a minor effect on increasing X-linked gene expression in the cloned pig embryos [16]. This was different from the result from knocking down YY1 in the cloned pig embryos. The differences in the ability to upregulate the expression of X-linked genes between the two methods may be related to the fact that knockdown of YY1 can inhibit both *XIST* transcription and *XIST* RNA localization on the X chromosome, whereas the knockdown of RLIM can only block *XIST* expression. The direct knockdown of *XIST* by siRNA also improved cloned embryo development, and this approach resulted in a higher cloning efficiency than the RNA interference of YY1 or RLIM [18]. The siRNA-mediated knockdown of YY1 or RLIM only inhibited the production of newly transcribed YY1 and RLIM mRNA but had no effect on the YY1 or RLIM protein that was already synthesized and maintained in the cloned embryos, which could still promote *XIST* expression. Thus, for improving the cloning efficiency, the direct knockdown of *XIST* was better than the indirect suppression of *XIST* expression by RNA interference of YY1 or RLIM.

## 4. Materials and Methods

### 4.1. Ethics Statement

This study was conducted following the “Guiding Opinions on the Care of Laboratory Animals” promulgated by the Ministry of Science and Technology of China. The protocol for animal experiments was approved by the Institutional Animal Care and Utilization Committee of South China Agricultural University. All efforts were made to minimize the suffering of the animals tested.

### 4.2. Design, Synthesis, and Transfection of siRNA

One siRNA that targeted the mRNA of the porcine *YY1* gene (XM_021099699) was designed.

The sequences of the anti-YY1 siRNA and NC siRNA are as follows:

YY1-siRNA (sense): CCGAGUACAUGACAGGAAATT
YY1-siRNA (antisense): UUUCCUGUCAUGUACUCGGTT
NC-siRNA (sense): UUCUCCGAACGUGUCACGUTT
NC-siRNA (antisense): ACGUGACACGUUCGGAGAATT

Anti-YY1 siRNA and NC siRNA were synthesized by GenePharma (Shanghai, China).

Porcine fetal fibroblasts (PFF) were cultured in Dulbecco’s modified Eagle’s medium (DMEM) complete medium containing 12% fetal bovine serum (Gibco) in a 10 cm diameter petri dish at 37 °C and 5% CO_2_. The cells were digested with trypsin after they were 90% confluent. siRNA transfection was performed using the transfection reagent Lipofectamine™ RNAiMAX (Invitrogen) according to the instructions. Forty-eight hours post-transfection, cells were collected for total RNA extraction and were stored at −80 °C for later use.

### 4.3. Real-Time Quantitative Polymerase Chain Reaction (qPCR) Analysis

Total RNA was extracted from PFF and microinjected embryos using the E.Z.N.A.^®^ Total RNA Kit II and Qiagen AllPrep DNA/RNA Micro Kit (Qiagen; Gaithersburg, MD, USA), respectively, according to the manufacturer’s instructions. The resulting total RNA was reverse transcribed using the PrimeScript™ RT reagent Kit with gDNA Eraser. The synthesized complementary DNA (cDNA) was detected by qPCR using the PowerUp™ SYBR™ Green Master Mix. GAPDH was used as the internal reference gene. The primers and their sequences are mentioned in Table 4. PCR reactions were performed using a QuantStudio 7 Flex system (Thermo Fisher Scientific Waltham, MA, USA) according to the manufacturer’s recommended parameters. The relative expression of genes was calculated using the 2^−∆∆Ct^ method.

### 4.4. Chromatin Immunoprecipitation-Sequencing (ChIP-Seq) and Data Analysis

ChIP was performed using the Bersin Bio Chromatin immunoprecipitation Kit according to the instructions. After the digestion of PFF, cells were cross-linked using 1% formaldehyde for 10 min at room temperature. Cross-linked cells were treated with glycine at room temperature for 5 min, centrifuged at 1000 rpm for 5 min at 4 °C, and rinsed twice with PBS. Chromatin fragments were generated by shearing cell lysates through sonication in 1% SDS lysis buffer. The sonicated sample was centrifuged at 20,000 g at 4 °C for 10 min, and the supernatant was transferred to a fresh centrifuge tube. Magnetic beads were prepared in advance by overnight incubation with YY1 antibody (Proteintech, Rosemont, IL, USA) and IgG antibody (Proteintech, Rosemont, IL, USA). The ultrasonically lysed samples were mixed with the antibody-incubated magnetic beads and incubated for 2–4 h. The DNA on the magnetic beads was eluted, and RNase A and Proteinase K were added sequentially to remove RNase and protease from the elute. This DNA was purified using phenol–chloroform extraction and sequenced by Novogene Corporation (Beijing, China) using the Illumina high-throughput sequencing platform NovaSeq 6000.

FastQC and Bowtie2 were used for the quality control and sequence alignment of the sequencing data [28]. Peaks calling was analyzed by the Model-based Analysis of ChIP-seq 2 (MACS2) software with parameters set to *p* < 0.005 [29]. Peaks were identified by MACS2, and their regions were annotated using the R package ChIPseeke [30]. The ChIP-seq IP data with the ChIP-Seq input data as a control were normalized using the bamCompare tool in the deepTools software based on the log2 fold difference in RPKM as a pattern, and a bigwig file was generated for visualization [31]. The multiBigwigSummary tool of deepTools was used to summarize the genome coverage of each sample bigwig file. The plotPCA tool of deepTools was used to perform the principal component analysis (PCA) of the data. The plotHeatmap and plotProfile tools were used to draw the upstream and downstream 2000 bp ChIP-seq data distribution heatmap and profile plot of signal enrichment (with the transcriptional start site as the center). The HOMER software was used to detect significantly enriched known motifs in identified peaks [32].

### 4.5. Analysis of the Binding Site of YY1 on the XIST Gene

The *XIST* gene sequences of each species were downloaded from the National Center for Biotechnology Information website. The binding sites of YY1 on the *XIST* gene of each species were searched on the MEME Suite website (https://meme-suite.org/meme/ (accessed on 22 November 2021)).

### 4.6. Production of Cloned Embryos

Fresh gilt ovaries were collected from local slaughterhouses, placed in normal saline (containing 1% penicillin and streptomycin sulfate), preheated to 37 °C, and transported to the laboratory within 2–3 h. The follicular fluid containing cumulus–oocyte complexes (COCs) was aspirated from antral follicles using a 10 mL syringe equipped with a needle. COCs with homogeneous cytoplasm and at least three layers of cumulus cells were selected, transferred to the oocyte maturation medium, and then cultured at 38.5 °C in a cell culture incubator with 5% CO_2_ for 42–44 h. After in vitro maturation, COCs were transferred into 200 µL of DPBS-PVA containing 1.0 mg/mL hyaluronidase to remove the surrounding cumulus cells by gentle pipetting. For the subsequent SCNT, mature oocytes with intact cell membranes, clear perivitelline spaces, and clearly visible extruded polar bodies were selected. The selected oocytes were fixed using a fixation needle and enucleated by blind suction. A single donor cell (*Duroc boar*) was injected into the perivitelline space of the enucleated oocytes. The reconstituted embryos were cultured in porcine zygote medium 3 (PZM-3) for 1 h and then transferred to an electrofusion solution with a direct current pulse of 100 v/mm, 100 μs, and 2DC to induce fusion. The reconstituted embryos were transferred to fresh PZM-3 and further cultured. After activation, the cleavage embryos and blastocysts were counted at 48 h and 144 h, respectively.

### 4.7. Microinjection of siRNA into Cloned Embryos

Different concentrations of siRNA (10 pL) were microinjected into the activated cloned embryos using a micropipette driven by a Piezo (PiezoXpert). Following microinjection, the embryos were cultured in PZM-3 medium at 38.5 °C and 5% CO_2_ in a humidified atmosphere.

### 4.8. Immunofluorescence Assay

Embryos were collected, rinsed gently thrice in DPBS-PVB, and fixed with 4% paraformaldehyde (Biosharp Biotechnology Company) for 15 min at room temperature. The fixed embryos were then permeabilized with 0.2% Triton X-100 for 15 min and treated with a blocking solution for 1 h; both procedures were performed at room temperature. The embryos were then incubated overnight with YY1 antibody (1:500; Proteintech) at 4 °C. The next day, the embryos were transferred to a diluted fluorescent secondary antibody (1:500; Thermo Fisher Scientific) and incubated at 37 °C for 1 h. The genomic DNA in the embryos was stained with DAPI (1:1000; Thermo Fisher Scientific) for 5 min and observed by fluorescence microscopy.

### 4.9. Embryo Transfer

Embryos at the 2-cell stage were selected and loaded into transfer tubes 22–26 h after activation. The selected embryos were transported to a pig farm with a portable incubator (Minitube, Delavan, WI, USA). Within 40–46 h prior to embryo transfer, estrous-synchronized Yorkshire sows were used as recipients. Anesthesia was induced with ketamine (25 mg/kg body weight) and xylazine (1.1 mg/kg body weight), respectively, and maintained with 3% isoflurane. The ovaries and oviducts of sows were surgically exposed. A syringe was used to deliver the cloned embryos to the oviducts of sows. Ultrasound examination was performed to monitor the pregnancy status of the recipient sows at 1 mo after the embryo transfer. The recipients gave birth to cloned piglets through natural birth. If natural farrowing did not occur until gestation day 116, the recipient sows were injected with a prostaglandin analog (cloprostenol; 200 g/recipient) to induce farrowing, and the farrowing conditions of the sows were recorded.

### 4.10. RNA Sequencing and Bioinformatics Analysis

Cloned embryos were collected at the 4-cell stage, and five to six embryos were mixed as one sample for sequencing. The cDNA was generated and amplified for sequencing based on the smartseq2 method. Library quality was assessed using the Agilent Bioanalyzer 2100 system. The clustered library preparations were then sequenced on an Illumina Nova platform, and 150 bp paired-end reads were generated. Clean read data were mapped to the Sus scrofa 11.1 reference genome. Differentially expressed genes (DEGs) between the YY1-siRNA and NC-siRNA groups were analyzed using the R package DEseq2 [33]. Gene Ontology (GO) analysis and Kyoto Encyclopedia of Genes and Genomes (KEGG) analyses were performed with the R package clusterProfiler [34].

### 4.11. Statistical Analysis

Data were analyzed using SPSS software version 20. The differences in values from more than two groups were analyzed by one-way analysis of variance. The difference in values between two groups was analyzed by Student’s *t*-test.

## 5. Conclusions

The RNAi-mediated knockdown of YY1 expression suppressed *XIST* transcription, upregulated X-linked gene expression, and improved the developmental efficiency of cloned pig embryos. This study not only indicated the involvement of YY1 in the regulation of *XIST* expression and XCI in cloned pig embryos but also established a new approach to enhance the developmental competence of cloned pig embryos. These findings will help to promote the development and application of the pig cloning technique.

## Figures and Tables

**Table 1:** Effects of the injection of YY1 siRNA on the in vitro developmental efficiency of porcine SCNT embryos.

Groups	No. of Repetition	No. of Cultured SCNT Embryos	No. of Cleaved Embryos (%, Mean)	No. of Blastocysts (%, Mean)
YY1-siRNA (10 μM)	2	83	67 (80.75 ± 3.08) ^ab^	24 (28.92 ± 0.49) ^ab^
YY1-siRNA (50 μM)	2	65	56 (86.08 ± 6.83) ^b^	21 (32.24 ± 5.83) ^b^
NC-siRNA (50 μM)	2	75	49 (65.33 ± 0.65) ^a^	17 (22.65 ± 1.46) ^a^

Values in the same column labeled with different superscripts differ at *p* < 0.05.

**Table 2:** Effects of injection of 50 µM YY1 siRNA on the in vitro developmental efficiency of porcine SCNT embryos.

Groups	No. of Repetition	No. of Cultured SCNT Embryos	No. of Cleaved Embryos (%, Mean)	No. of Blastocysts (%, Mean)
YY1-siRNA (50 μM)	8	305	257 (84.29 ± 5.05) ^b^	81 (26.85 ± 6.76) ^b^
NC-siRNA (50 μM)	8	318	229 (71.74 ± 5.21) ^a^	60 (18.58 ± 6.54) ^a^

Values in the same column labeled with different superscripts differ at *p* < 0.05.

**Table 3:** Effects of injection of YY1 siRNA on the in vivo developmental rate of porcine sSCNT embryos.

Groups	No. of Total/Farrowed Recipients	No. of Transferred Cloned Embryos	No. of Total Born Cloned Piglets	No. of Live-Born Cloned Piglets
YY1-siRNA (50 μM)	6/2	1271	8	3
NC-siRNA (50 μM)	5/0	971	0	0

**Table 4:** Primers used for the quantitative polymerase chain reaction.

Gene	GenBank Accession No.	Primer Sequence
*YY1*	XM_021099699	(forward): ATACCCGGCATCGACCTCTC
		(reverse): AACATCTTTGTGCAGCCTTTGT
*XIST*	KC753465	(forward): CTTGCCGCAATCGAAAACAT
		(reverse): ACCAATTCCACCACCCTTTC
*LOC100622504*	XM_003360396	(forward): GTGAGACAACGTGCTCTGAGA
		(reverse): ATTCCATGGGTTCCTGAGCTG
*LOC110257861*	XR_002340890	(forward): GCACAGTACCTTCTCCCCTTC
		(reverse): CCCTGTTGCTTTGGTTTCGAG
*RBM3*	NM_001243419	(forward): GCTTCAAATGCAATGAGAGCCA
		(reverse): TCTGCCATTTCCATAGCCCTG
*H2AB2*	XM_021080443	(forward): AAAAGGAGCCGTCGAAAGTCA
		(reverse): GTCAGGTACTCGATGGTGGC
*HAUS7*	XM_021079955	(forward): CTCTGACTTCCACCAGCTCATC
		(reverse): CTCTTCCATTTTGCTGGTTGGC
*GAPDH*	NM_001206359	(forward): TGACCCCTTCATTGACCTCC
		(reverse): CTCCGCCTTGACTGTGCC

## Data Availability

The data that support this study are available within the article and available from the authors upon request.

## Abbreviation

bp	Base pair
BP	Biological process
CC	Cellular component
cDNA	Complementary DNA
ChIP-seq	Chromatin immunoprecipitation sequence
COCs	Cumulus oocyte complexes
DEGs	Differentially expressed genes
DMEM	Dulbecco’s Modified Eagle Medium
DNA	Deoxyribonucleic acid
DPBS	Dulbecco’s Phosphate Buffered Saline
FBS	Fetal bovine serum
GAPDH	Glyceraldehyde-3-phosphate dehydrogenase
GO	Gene Ontology
KEGG	Kyoto Encyclopedia of Genes and Genomes
mRNA	Messenger RNA
MF	Molecular function
PCA	Principal component analysis
PEF	Porcine embryonic fibroblast
PZM-3	Porcine zygote medium 3
qPCR	Quantitative Real-time PCR
RLIM	Ring finger protein, LIM domain interacting
RNA	Ribonucleic acid
RPKM	Reads per kilobase per million mapped reads
SCNT	Somatic cell nuclear transfer
sgRNA	Single guide RNA
siRNA	Small interfering RNA
TES	Transcription termination site
TSS	Transcriptional start site
XCI	X-chromosome inactivation X
XIST	X-inactive specific transcript
YY1	Yin yang 1
